# The association between the monocyte Chemoattractant protein-1–2518A/G polymorphism and the risk of tuberculosis in the Chinese population: A meta-analysis and systematic review

**DOI:** 10.1016/j.clinsp.2025.100685

**Published:** 2025-05-11

**Authors:** Hongfang Lu, Jingang Wang, Xinyu Song, Xiaoqi Xiong

**Affiliations:** a The First Clinical College of China Three Gorges University, Yichang, PR China; b Department of Respiratory and Critical Care Medicine, Yichang Central People's Hospital, Yichang, PR China; c Department of Hematology, Yichang Central People's Hospital, Yichang, PR China

**Keywords:** Meta-Analysis, Monocyte Chemoattractant Protein-1, Tuberculosis

## Abstract

**Objectives:**

No meta-analysis has been conducted on the relationship between the MCP-1–2518A/G gene polymorphism and tuberculosis susceptibility in the Chinese population. The aim of this study was to investigate whether MCP-1–2518A/G polymorphism is associated with susceptibility to TB in Chinese.

**Method:**

Publications addressing the association between the MCP-1–2518A/G polymorphism and TB risk were selected from the PubMed, Embase, CNKI and Wanfang databases. Data were extracted from the studies by two independent reviewers. Statistical analysis was performed using RevMan 5.1 and STATA 12 software.

**Results:**

Ten case-control studies were extracted from 10 articles on polymorphisms involving 1634 TB patients and 1768 controls. The results of the meta-analysis showed that a significantly increased risk for TB was found for Chinese individuals with the polymorphism as follows: dominant model (GG+GA vs. AA Odds Ratio [OR = 1.53], 95 % CI 1.14‒2.07, *p* = 0.000); recessive model (GG vs. GA+AA Odds Ratio [OR = 1.63], 95 % CI 1.13‒2.35, *p* = 0.009); and a homozygote comparison (GG vs. AA Odds Ratio [OR = 1.93], 95 % CI 1.19‒3.13, *p* = 0.008). In the subgroup analysis by ethnicity, significantly elevated risks were found in non-Han Chinese individuals but not in Han Chinese individuals (mutant homozygous GG vs. AA: OR = 3.81, 95 % CI 2.07‒7.00, *p* = 0.000).

**Conclusions:**

This meta-analysis suggested that the -2518A/G polymorphism of the MCP-1 gene is a risk factor for TB in Chinese individuals. In the GG vs. AA model, non-Han Chinese individuals had a higher risk of tuberculosis than Han Chinese individuals.

## Highlights

• This meta-analysis investigated MCP-1–2518A/G polymorphism in Chinese population.
• The MCP-1–2518A/G polymorphism increased tuberculosis susceptibility.
• Non-Han Chinese individuals showed a higher risk of tuberculosis.

## Introduction

Tuberculosis (TB) is one of the most important infectious causes of death worldwide.^1^ According to the WHO 2020 Global Tuberculosis Report, China had the second largest number of incident TB cases, accounting for 9 % of the global burden.^2^ Although 1.7 billion people globally are estimated to be infected with *Mycobacterium tuberculosis*, only some of these people will go on to develop active tuberculosis,^3^ indicating that the individuals have different TB susceptibility. Although the underlying etiological mechanism of TB infection is still unclear, host genetic genes are considered to be one of the reasons for the incidence of tuberculosis and may impact the disease before its onset. In recent years, genetic variants, including Epiregulin (EREG),^4^ Toll-Like Receptor-2 (TLR2)^5^ and Nuclear Factor-Erythroid-2 (NF-E2)-related Factor-2 (Nrf2),^6^ have been demonstrated to be associated with TB susceptibility, but their roles in the incidence of TB are conflicting. Among various cytokines, MCP-1, also referred to as chemokine C—C motif ligand-2 (CCL2), is a member of the small inducible gene family. The gene encoding MCP-1 is located in the 17q11.2-q12 chromosomal region; single-nucleotide polymorphisms such as −2518A/G (rs1024611) and −362G/C (rs2857656) have been identified. These polymorphisms may influence MCP-1 secretion, impacting TB susceptibility.^7^ Several studies have reported the association between MCP-1 polymorphisms and TB susceptibility, but the results were inconsistent due to limited sample sizes and different study populations. In addition, the association between the MCP-1–2518A/G gene polymorphism and susceptibility to tuberculosis has not been reported in the Chinese population.^8^ Therefore, the authors performed a systematic review and meta-analysis in this article to summarize the associations between the MCP-1–2518A/G gene polymorphism and TB susceptibility in Chinese individuals.

## Materials and methods

A systematic literature search in the PubMed, Embase, Wanfang (www.Wanfangdata.com.cn) and CNKI databases (China National Knowledge Infrastructure, www.cnki.net) was carried out to identify studies involving the association between TB risk and MCP-1 polymorphisms on March 1, 2023. The search terms were as follows: ‘TB or tuberculosis’ in combination with ‘polymorphism or variant or mutation’ and ‘Monocyte Chemoattractant Protein-1 or MCP-1 or CCL2 or Chemokine (C—C motif) ligand 2′. The languages were limited to English and Chinese. Ethical approval was waived because this article does not contain any studies with human or animal subjects performed by any of the authors.

### *Inclusion and exclusion criteria*

The inclusion criteria were defined as follows: a) Studies that evaluated the association between MCP-1–2518A/G and TB risk; b) Studies with a case-control study design based on unrelated individuals; c) Studies with sufficient data (genotype distributions for cases and controls) available to estimate an Odds Ratio (OR) with its 95 % CI; and d) Studies with genotype distributions in the control group that were consistent with Hardy Weinberg Equilibrium (HWE). Studies were excluded if one of the following existed: a) The study design was based on family or sibling pairs; b) The genotype frequencies or number not reported; c) The studies were reviews or abstracts; or d) The studies included HIV/TB patients. If more than one study by the same authors using the same case series was published, either the study with the largest sample size or the one that was published most recently was included. The supporting PRISMA checklist is available as supporting information.

### *Data extraction*

Two investigators independently extracted the data and reached a consensus for all items. If the 2 investigators generated different results, they checked the data again and discussed until they came to an agreement. If they could not reach an agreement, an expert was invited to the discussion. Data extracted from the selected articles included the first author’s name, year of publication, sample ethnicity, and genotype number in the cases and controls.

### *Statistical analysis*

Before assessing the effects of MCP-1 polymorphisms on TB susceptibility, the authors tested whether the genotype frequencies of controls were in HWE by using the χ^2^ test. The authors also quantified the effect of heterogeneity by the I^2^ test. When a significant *Q* test (*p* < 0.1) or I^2^ > 50 % indicated heterogeneity across studies, the random effects model was used; otherwise, the fixed effects model was used.^8^ The Odds Ratio (OR) and 95 % Confidence Interval (95 % CI) were employed to estimate the risk of TB with polymorphisms of the MCP-1 gene. A χ^2^-test-based *Q* statistic test was performed to assess the between-study heterogeneity.^9^ An analysis of sensitivity was performed to evaluate the stability of the results. Finally, potential publication bias was investigated using Egger’s regression test.^10^ A *p* < 0.05 was regarded as statistically significant. Meta-analysis was performed by Review Manager 5.1 and Stata 12 (Stata Corporation, College Station, Texas, USA) software packages.

## Results

### *Eligible studies*

**Fig. 1:**
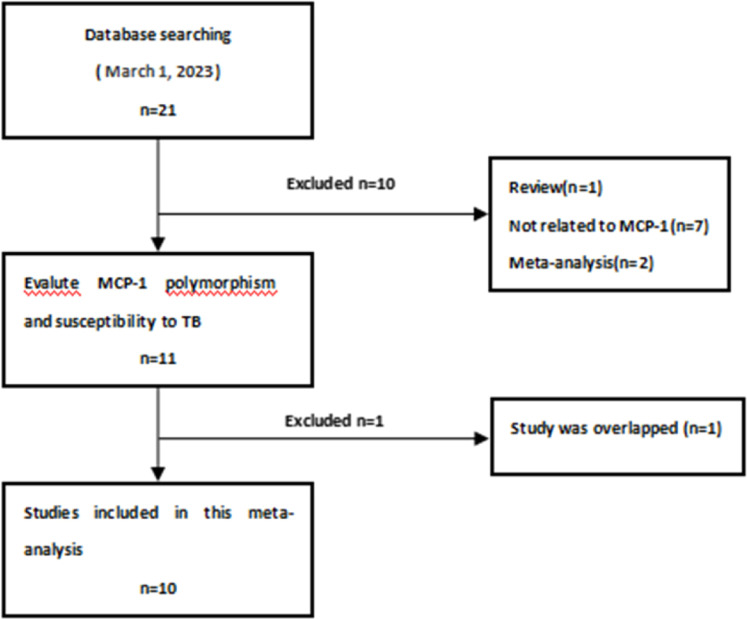
The process for the screening of 10 potentially relevant studies included in the meta-analysis. MCP-1, Chemokine (C—C Motif) ligand 2, TB, Tuberculosis.

The search strategy retrieved 21 potentially relevant studies. According to the inclusion criteria, 10 studies with full texts were included in this meta-analysis. The flow chart of study selection is summarized in Fig. 1. As shown in Table 1, there were 10 case-control studies with 1634 TB patients and 1768 controls concerning the MCP-1–2518A/G polymorphism. For the 2 ethnicities addressed, 8 studies focused on China's Han ethnic group^11^, ^12^, ^13^, ^14^, ^15^, ^16^, ^17^, ^18^ and 2 focused on the non-Han population of China.^19^^,^^20^ The authors performed subgroup analysis for the Han and non-Han groups.

**Table 1:** Pooled data for MCP-1 2518A/G analysis.

**Author**	**Year**	**Ethnicity**	**Case**				**Control**				**HWE**
			**GG**	**GA**	**AA**	**Total**	**GG**	**GA**	**AA**	**Total**	**p-value**
Chen, Tong, Li, Wu and Zhang^11^	2015	Han Chinese	56	57	23	136	40	77	35	152	0.860
G.H. Cheng^12^	2018	Han Chinese	66	90	27	183	97	82	31	210	0.053
Chu, Tam, Wong, Kam, Lau and Chiang^13^	2007	Han Chinese	110	200	93	403	113	233	115	461	0.816
Feng, Mokrousov, Wang, Nelson, Jiao, Wang, Sun, Zhou, Xiao, Gu, Wu, Ma and Shen^14^	2011	Han Chinese	106	157	38	301	117	170	51	338	0.400
Shi, Yang, Sun, Yin and Song^15^	2015	Han Chinese	82	187	129	386	102	189	95	398	0.689
Xu, Xie, Chen, Xing, Zhang, Li and Zhu^16^	2009	Han Chinese	36	49	15	100	14	45	41	100	0.770
Yang, Zhuang, Li, Zhang and Song^29^	2009	Han Chinese	84	62	21	167	42	83	42	167	0.938
Qinyan Yang^19^	2014	Non-Han Chinese	42	12	6	60	18	30	12	60	0.938
Baoqin Zhang^17^	2009	Non-Han Chinese	49	76	16	141	35	77	40	152	0.860
Zhang^20^	2013	Han Chinese	56	57	23	136	40	77	35	152	0.860

HWE, Hardy Weinberg Equilibrium.

### *Quantitative synthesis*

**Fig. 2:**
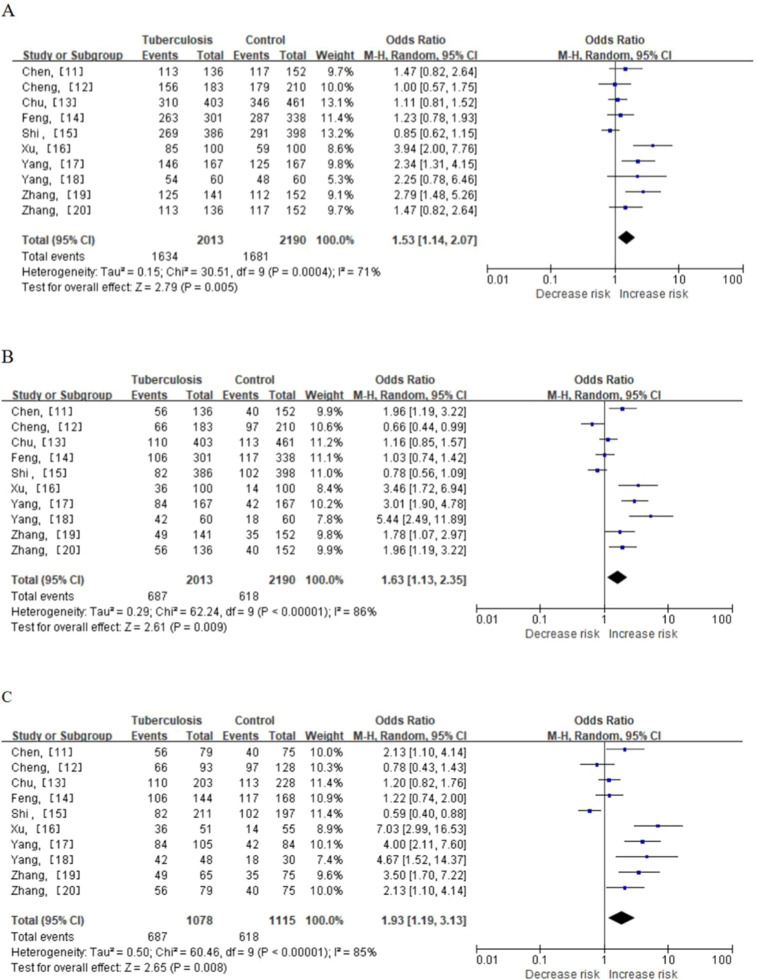
Meta-analysis of the association between MCP-1–2518A/G polymorphism and susceptibility to TB. (A) GG+GA vs. AA. (B) GG vs. GA+AA. (C) GG vs. AA. MCP-1, Chemotactic Chemokine (C—C Motif) ligand 2, TB, Tuberculosis, AA, Wild-type homozygous, GG, Mutant Homozygous, GA, Mutant Heterozygous.

**Fig. 3:**
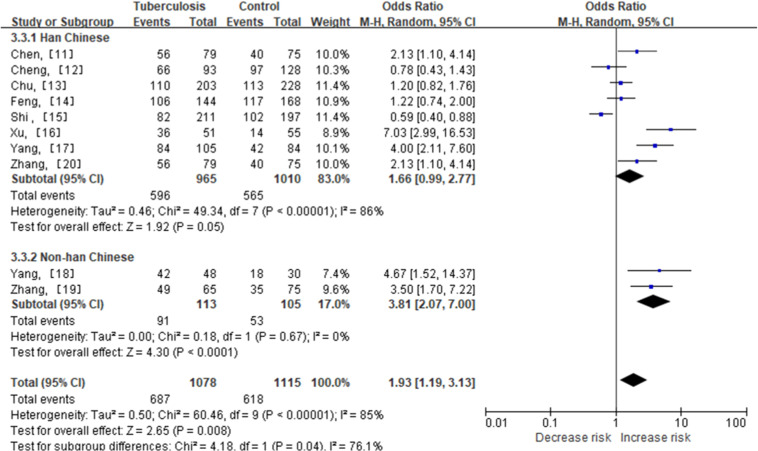
Subgroup analysis of MCP-1–2518A/G polymorphism by ethnicity (GG vs. AA). TB, Tuberculosis; 95 % CI, 95 % Confidence Interval; df, degrees of freedom; MCP-1, CC Chemokine ligand 2; TB, Tuberculosis.

The analysis of the MCP-1–2518A/G polymorphism, qualified in 10 studies (Table 1), revealed that the MCP-1–2518A/G polymorphism increased the risk of tuberculosis in Chinese people (dominant model (GG+GA vs. AA OR = 1.53, 95 % CI 1.14‒2.07, *p* = 0.000), recessive model (GG vs. GA+AA OR = 1.63, 95 % CI 1.13‒2.35, *p* = 0.009), and homozygote comparison (GG vs. AA OR = 1.93, 95 % CI 1.19‒3.13, *p* = 0.008)) (Table 2, Fig. 2). Because the Chinese population includes Han Chinese and ethnic minorities, the authors compared the differences between the two groups. In the subgroup analysis, an increased risk was found in the non-Han Chinese population (mutant homozygous GG vs. AA: OR = 3.81, 95 % CI 2.07‒7.00, *p* = 0.000) (Table 3, Fig. 3). Sensitivity analysis was performed by the sequential omission of individual studies. The results showed that the estimated pooled OR was not changed, indicating that the present results were statistically robust. There was significant heterogeneity for the overall comparisons (dominant model: *p* = 0.000; recessive model: *p* = 0.000; homozygote comparison: *p* = 0.000). In the subgroup analysis by ethnicity, the results were similar in the Han population. In the non-Han population, there was significant heterogeneity for the recessive comparison (GG vs. GA+AA, *p* = 0.020) but not for the dominant comparison (GG+GA vs. AA, *p* = 0.730) or homozygous comparison (GG vs. AA, *p* = 0.670) (Table 2). *Q*-tests and I2 statistics were employed to test the heterogeneity among the selected publications. Heterogeneity was observed in all of the models. Thus, when a significant *Q*-test (*p* < 0.1) or I^2^ > 50 % indicated heterogeneity across the studies, the random effects model was used; otherwise, the fixed effects model was used (Table 2, Table 3).

**Table 2:** Pooled analysis for the associations between polymorphisms of MCP-1–2518A/G and the risk of TB.

	SNP	N	Test of association		Test of heterogeneity		Publication bias (Egger’s test)	
			Odds ratio	95 % CI	p*-*value	p-value	I^2^ (%)	p-value
−2518A/G	10	GG+GA vs. AA	1.53	1.14 – 2.07	0.005	0.000	71	0.016
		GG vs. GA+AA	1.63	1.13 – 2.35	0.009	0.000	86	0.006
		GG vs. AA	1.93	1.19 – 3.13	0.008	0.000	85	0.008

SNP, Single-Nucleotide Polymorphism; CI, Confidence Interval; I^2^, Statistical variable of heterogeneity test; MCP-1, Chemotactic chemokine (C—C motif) ligand 2; TB, Tuberculosis; AA, Wild-type homozygous; GG, Mutant homozygous.

**Table 3:** Subgroup analysis for the associations between the polymorphisms of MCP-1–2518A/G and the risk of tuberculosis.

SNP	Comparison	Subgroup	Test of association		Test of heterogeneity		Subgroup differences	
		Ethnicity	Odds ratio (95 % CI)	p-value	p-value	I^2^ (%)	p-value	I^2^ (%)
−2518A/G	GG+GA VS. AA	Han Chinese	1.40 (1.03‒1.90)	0.030	0.001	70	0.050	74.9
		Non-Han Chinese	2.64 (1.53‒4.54)	0.000	0.730	0		
	GG VS. GA+AA	Han Chinese	1.43 (0.98‒2.09)	0.060	0.000	85	0.210	36.1
		Non-Han Chinese	2.99 (1.00‒8.92)	0.050	0.020	82		
	GG VS. AA	Han Chinese	1.66 (0.99‒2.77)	0.050	0.000	86	0.040	76.1
		Non-Han Chinese	3.81 (2.07‒7.00)	0.000	0.670	0		

CI, Confidence Interval; I^2^, Statistical variable of heterogeneity test; MCP-1, Chemotactic Chemokine (C—C motif) ligand 2; AA, Wild-type homozygous; GG, Mutant homozygous, CG, Mutant heterozygous.

### *Publication bias*

**Fig. 4:**
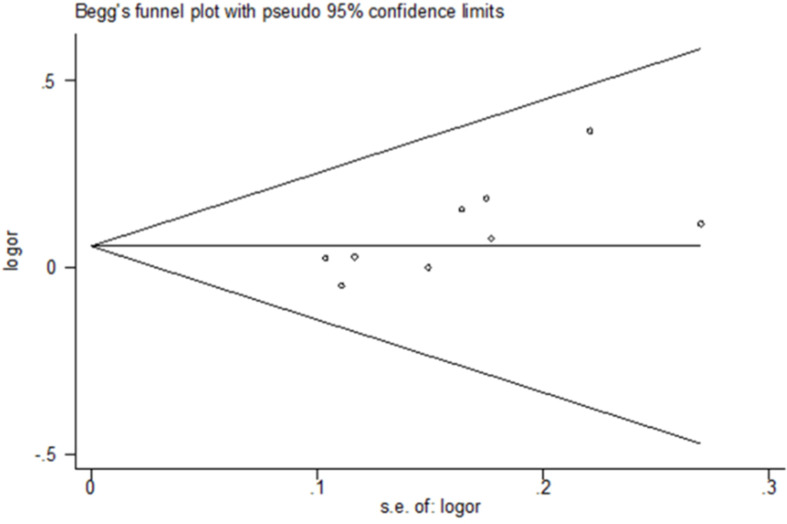
Begg’s funnel plot for publication bias in the selection of studies on the MCP-1–2518A/G polymorphism (GG+GA vs. AA).

The authors used Begg’s funnel plot and Egger’s test to address potential publication bias in the available literature. The publication bias of the meta-analysis on the association between the MCP-1–2518A/G polymorphism and TB susceptibility was detected. The shape of the funnel plots revealed some evidence of funnel plot asymmetry. Egger’s test also showed that there was statistical significance for the evaluation of publication bias (p-dominant model = 0.016, p-recessive model = 0.006, p-homozygote comparison = 0.008) (Table 2, Fig. 4).

## Discussion

Tuberculosis has been one of the most important illnesses in the history of the world, and it has not been understood why only some people, and not others, develop the disease. Recently, published Genome-Wide Association Studies (GWAS).^21^, ^22^, ^23^, ^24^, ^25^ demonstrated that host genetics strongly influence TB susceptibility. Since it acts as a chemokine for T-cells and monocytes/macrophages, MCP-1 was supposed to be a strong candidate for increasing TB susceptibility. Slight and Khader^26^ reported that macrophages can secrete MCP-1 after infection with *Mycobacterium tuberculosis*, and the MCP-1 gene polymorphism is closely related to the susceptibility of *Mycobacterium tuberculosis* in the plasma/serum of active tuberculosis patients.^26^ It was found that there was a significant correlation between 2518 G alleles of the MCP-1 promoter region and tuberculosis in Chongqing, China.^16^ Children, adults, and the general population with the GG genotype were 7 times, 5.1 times, and 6 times, respectively, more likely to develop TB than those with the AA genotype.^16^ At the same time, they found that the blood level of MCP-1 in people with the GG genotype was higher, which may be one of the important factors of TB susceptibility.^16^ Biswas et al.,^27^ reported that MCP-1 showed a positive correlation with IL-12, IFN-γ, and TNF-α, and a normal CCL2 level is essential for a normal Th1 response.^27^ The −2518 G allele produces less MCP-1 than the A allele, which leads to an improper Th1 response and makes the host susceptible to tuberculosis. However, a study with a smaller sample size in Brazil found that there was no correlation between the MCP-1 polymorphism and tuberculosis.^28^ Although the exact molecular mechanism is still unclear, several polymorphisms in MCP-1 have been reported previously,^21^, ^22^, ^23^^,^^26^, ^27^, ^28^ and the results are controversial.

The association between the MCP-1–2518A/G gene polymorphism and Tuberculosis (TB) susceptibility has become a topic of considerable interest in genetic epidemiology, and one of the most notable differences across the studies is the focus on ethnicity and its impacts on the observed associations. In the meta-analysis, the authors included a total of 10 case-control studies and evaluated the association of one common polymorphism in the MCP-1 gene with the risk of TB, specifically within the Han and non-Han Chinese populations. The pooled results indicated that there was a significant association between the MCP-1–2518A/G polymorphism and TB for all models: the dominant model (GG+GA vs. AA), recessive model (GG vs. GA+AA), and homozygote comparison (GG vs. AA). To avoid the heterogeneity of ethnicity, 10 eligible case-control studies were stratified into 2 subgroups (Han Chinese and non-Han Chinese). The authors found that the MCP-1–2518A/G GG genotype increased the risk of TB in the 2 subgroups by ethnicity analysis, which suggested a possible role of ethnic differences in genetic backgrounds and the environment in which they lived. However, because only 2 studies with a non-Han Chinese population were selected, these results should be interpreted with caution, and more studies are needed to further investigate this association.

The present findings suggest a significant association between the MCP-1–2518A/G polymorphism and TB susceptibility, particularly in non-Han Chinese patients. This aligns with previous studies that have examined similar relationships in broader populations, though the ethnic and geographic scope of the studies varies.^24^^,^^25^ Therefore, the ethnic heterogeneity within the Chinese population could account for the observed differences in risk, supporting the need for tailored genetic research that considers population-specific factors. Vásquez-Loarte et al.^25^ introduced a novel ethnic classification system based on genetic distance, migration, and linguistic origins, proposing that this approach may enhance the homogeneity of study populations.^25^ Their findings demonstrated that the association between the MCP-1 polymorphism and TB susceptibility was only significantly present in East Asian and Latin American populations,^25^ which suggests that limitations of conventional ethnic categories in genetic studies have appeared and should use a more refined classification system to reduce bias and improve the validity of the results. In contrast, Li et al.,^24^ focused on a broader dataset encompassing multiple ethnic groups, including Asians, Americans, Africans, and Europeans^24^; they showed a significant association between MCP-1 polymorphism and TB susceptibility in Asian and American populations, but no such associations were observed in African or European populations.^24^ These findings are consistent with these own results in the Chinese populations, while they also suggested ethnic variations in the association between MCP-1 polymorphisms and TB susceptibility. Therefore, a diverse range of ethnic groups would help uncover global patterns of TB susceptibility and inform public health strategies for diverse populations.

The authors attempted to minimize the likelihood of bias by developing a detailed protocol before initiating the study. Some insurmountable limitations of this meta-analysis may affect the results and even the subsequent conclusions. First, the number of published studies was not sufficiently large for a comprehensive analysis, and some studies with small sample sizes may not have enough statistical power to explore the real association. Second, the overall outcomes were based on individual unadjusted Ors; a more precise estimation should be adjusted by environmental and other confounding factors. Despite these limitations, the meta-analysis had several advantages. First, a substantial number of cases and controls were pooled from different studies, which significantly increased the statistical power of the analysis. Second, the quality of the case-control studies included in the current meta-analysis was satisfactory and met the inclusion criterion.

## Conclusion

In summary, the authors have shown that the MCP-1–2518A/G polymorphism increased TB susceptibility in the Chinese population, and those with the −2518A/G GG genotype appeared to have a higher risk of TB in the non-Han Chinese population. However, large and well-designed studies are warranted to validate these findings. As TB-related genetic factors may interfere with nongenetic risk factors, such as the environment, future studies should therefore be stratified accordingly. In addition, gene-gene and gene-environment interactions should also be investigated in the future.

## Authors’ contributions

Hongfang Lu wrote the draft. Hongfang Lu and Jingang Wang conceived the study and revised the manuscript. Xinyu Song and Xiaoqi Xiong collected the data and performed the statistical analysis. All authors read and approved the final manuscript.

## Declaration of competing interest

The authors declare no conflicts of interest.
